# Comparison of proteomic datasets from hypertrophic chondrocytes in response to ER stress

**DOI:** 10.1016/j.dib.2016.02.065

**Published:** 2016-03-02

**Authors:** Mateusz Kudelko, Rakesh Sharma, Kathryn S.E. Cheah, Danny Chan

**Affiliations:** a School of Biomedical Sciences, The University of Hong Kong, Pokfulam, Hong Kong, China; b Department of Biology and Chemistry, City University of Hong Kong, Tat Chee Avenue, Hong Kong, China

**Keywords:** Cartilage, Proteomics, Hypertrophic chondrocytes, ER stress

## Abstract

Cartilage proteomics is challenging due to the dominance of poorly soluble matrix components and limited available tissue. Using a “spatial” strategy coupled to MS/MS analysis we have specifically labeled and extracted hypertrophic chondrocytes within the growth plate providing thus a comprehensive proteomic map of normal hypertrophic chondrocytes. Furthermore our established 13del mouse model in which the activation of ER stress did not lead to apoptosis of the hypertrophic cells allowed us to address the natural consequences of ER stress in vivo. Thus our data provide also an overview of proteomic changes occurring in cells under ER stress. Associated with the published study [1] this dataset article provided the detailed information of experimental designing, methods, features as well as the raw data of mass spectrometry (MS) identification. Furthermore the data presented here allow the reader to assert the extent of proteomic changes occurring under ER stress in hypertrophic chondrocytes as well as address the data technical reproducibility in both wild type and stress condition. The mass spectrometry proteomics data can be fully accessed from the ProteomeXchange Consortium via the PRIDE partner repository with the dataset identifier *PXD002125*.

**Specifications Table**

**Table:** 

Subject area	*Biology*
More specific subject area	*Cartilage proteomics*
Type of data	*Table, Excel Files*
How data was acquired	*LC-MS/MS using an UltiMate 3000 HPLC system (Dionex) connected online with an LTQ-Orbitrap Velos (Thermo Scientific)*
Data format	*Analyzed*
Experimental factors	*Hypertrophic chondrocytes under normal or ER stress environment*
Experimental features	*Proteins were reduced, alkylated before being in solution digested. For each sample protein concentrations were estimated using the linear Bradford assay after specific removal of detergent using Pierce detergent removal resin and columns.*
Data source location	*Hong Kong, China*
Data accessibility	*The mass spectrometry proteomics data have been deposited to the ProteomeXchange Consortium*[2]*via the PRIDE partner repository with the dataset identifier PXD002125.*

**Value of the data**

• Using an innovative approach we developed a “spatial” strategy that allow us to dissect GFP marked hypertrophic chondrocytes from the growth plate cartilage providing thus a specific proteomic data of these cells.
• Our data describe proteomic changes occurring in cells under ER stress.
• Comparison of proteomic data augment our understanding of growth plate pathologies caused by protein misfolding in the pathogenesis of chondrodysplasias.

## 1 Data, experimental design, materials and methods

The data shown here present Supplementary Table 1 in which a coefficient of variance (CV) for the independent sample runs (*n*=5) for both the wt and 13del condition was calculated. Compared to the previous data [1], the following table allows the reader to assert the technical reproducibility between sample runs for each condition (wt and 13del). Based on previously published article [1], three of the most representative proteins, Col10a1, Hspa5 and Vdac1 respectively, were chosen. A box and whisker plot showing the normalized spectral counts calculated by Scaffold in wt (*n*=5) and 13del (*n*=5) is presented (Supplementary Fig. 1) as an example to illustrate the technical variability of sample runs. Furthermore a fold change and *p*-value based on Fisher’s Exact test is presented to depict the extent of proteomic changes under ER stress (Supplementary Table 1).

### 1.1 Experimental design

eGFP labeled hypertrophic chondrocytes were specifically extracted from distal and proximal tibia of P10 wt-*Col10a1-eGFP* and 13del;*Col10a1-eGFP* mice. After the proteins were extracted and peptides digested, samples were analyzed in triplicate by LC-MS/MS (LTQ-Orbitrap Velos). Altogether five biological replicates per genotype were used for analysis.

### 1.2 LC–MS/MS and data analysis

Tryptic peptides were loaded onto a self-packed PicoTip^®^ column (360 μm outer diameter, 75 μm inner diameter, 15 μm tip, New Objective) packed with 10 cm length of C18 material (ODS-A C_18_ 5 μm beads, YMC) with a high-pressure injection pump (Next Advance) at 1 μl/min in 98% solvent A (0.1% (v/v) formic acid) and 2% solvent B (0.1% (v/v) formic acid in acetonitrile) for 6 min before being eluted with a linear gradient B from 2% to 40% for 120 min at a flow rate of 300 nL min^−1^. The LTQ-Orbitrap Velos was controlled using Xcalibur, version 2.0.7 (Thermo Fisher Scientific) and operated in data-dependent acquisition mode whereby the survey scan was acquired in the Orbitrap with a resolving power set to 60,000 (at 400 m/z). MS/MS fragment ions were identified in LTQ mass analyzer and for each duty cycle the 20 most intense precursor ions were selected for MS/MS. We used ion injection times of 350 ms and 150 ms for the MS and MS/MS scans with an automatic gain control targets for MS (FT) and MS/MS (LTQ) set to 1 million and 10,000 respectively. The mass range for precursor ion selection was set between 350 m/z and 1700 m/z. CID mode was used to carry fragmentation with following parameters: 35% normalized collision energy, activation time set to 0.25 and 10 ms. Ions selected for fragmentation were dynamically excluded for a period of 30 s from MS/MS analysis. For MS/MS analysis we selected specifically monoisotopic precursor mass and rejected singly charged ions. The lock mass was enabled for accurate mass measurements using Polydimethylcyclosiloxane (m/z, 445.12) ions.

The acquired MS/MS data were converted to Mascot Generic format using Proteome Discoverer 1.3 software (Thermo Fisher Scientific). We used MASCOT version 2.2.06 (Matrix Science) against UniProtKB mouse database of canonical sequences (Oct 2014; 16,480 entries) to analyze all acquired data. Enzyme specificity was set to trypsin with a maximum of two missed cleavages; S-carboxamidomethylation of cysteine residues specified as a fixed modification and oxidation of methionine specified as variable modification. Parent ion and fragment ion mass tolerance were set to 10 ppm and 0.5 Da, respectively. The Mascot search results (.dat files) were incorporated into Scaffold 4 (version 4.4.3, Proteome Software). Peptide-spectrum matches were accepted if the peptide was assigned a probability >0.95 as specified by the Peptide Prophet algorithm [3]. Protein identifications were accepted if the protein contained at least two unique peptide counts and the protein was assigned a probability >0.99 by the Protein Prophet algorithm and the FDR was<1%. We excluded from the analysis contaminants, such as keratin or immunoglobulin.
