# Supplementary material for: Comparison of proteomic datasets from hypertrophic chondrocytes in response to ER stress
Source: Data Brief. 2016 Mar 2;7:449–51. doi: 10.1016/j.dib.2016.02.065 (PMC4789308; doi:10.1016/j.dib.2016.02.065)
Supplement: Supplementary file 2 — Supplementary material [file mmc2.pptx]

## Slide 1
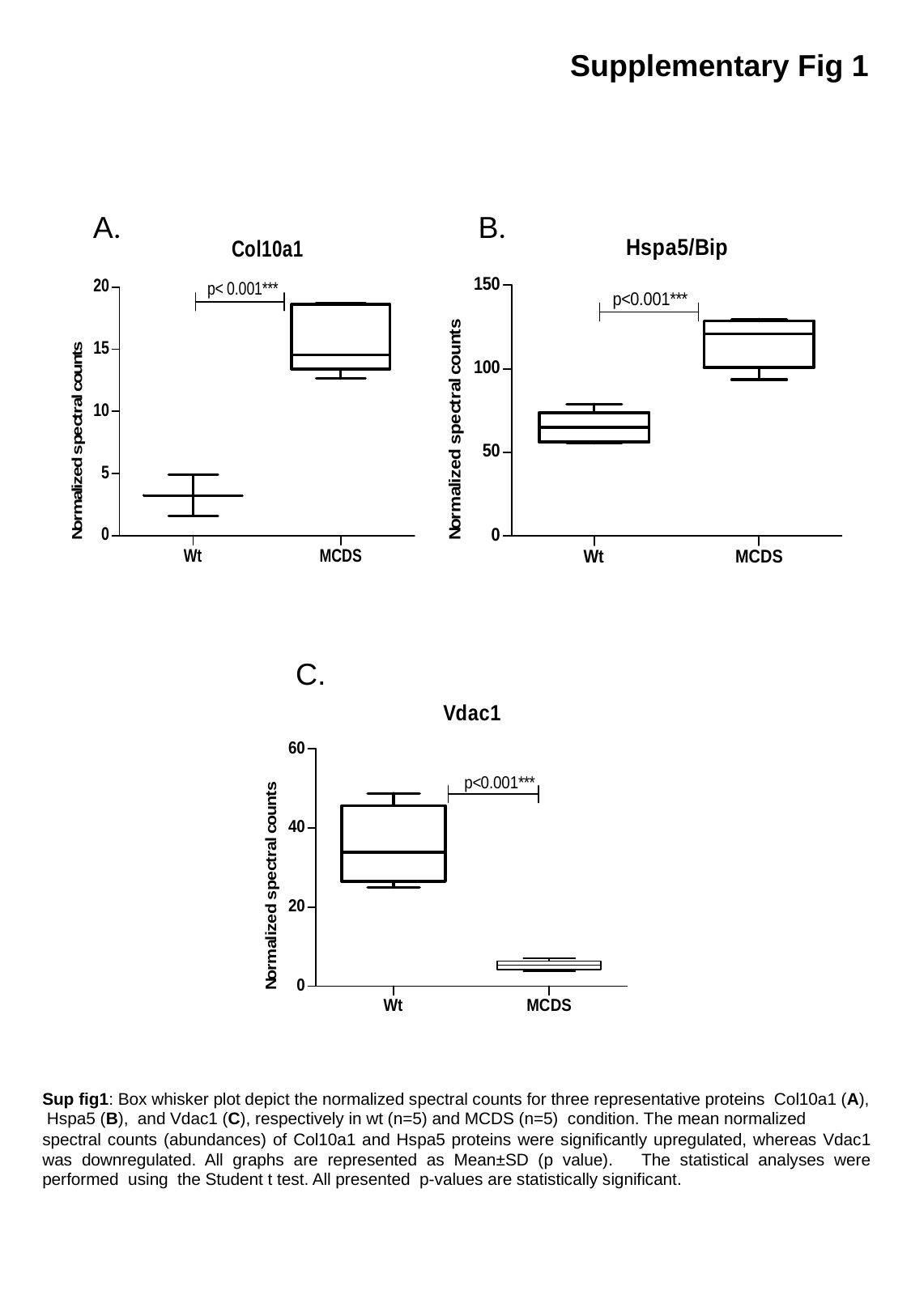

Supplementary Fig 1
A.
B.
C.
Sup fig1: Box whisker plot depict the normalized spectral counts for three representative proteins Col10a1 (A),
 Hspa5 (B), and Vdac1 (C), respectively in wt (n=5) and MCDS (n=5) condition. The mean normalized
spectral counts (abundances) of Col10a1 and Hspa5 proteins were significantly upregulated, whereas Vdac1 was downregulated. All graphs are represented as Mean±SD (p value). The statistical analyses were performed using the Student t test. All presented p-values are statistically significant.
